# Barriers to Mindfulness: a Path Analytic Model Exploring the Role of Rumination and Worry in Predicting Psychological and Physical Engagement in an Online Mindfulness-Based Intervention

**DOI:** 10.1007/s12671-017-0837-4

**Published:** 2017-11-06

**Authors:** Moitree Banerjee, Kate Cavanagh, Clara Strauss

**Affiliations:** 1 School of Psychology, University of Sussex, Pevensey Building, Falmer, BN1 9QH UK; 2 Sussex Mindfulness Centre, Worthing, UK; 3 Sussex Partnership NHS Foundation Trust, Worthing, UK

**Keywords:** Engagement, Mindfulness, Perseverative thinking, Rumination, Worry, Self-help, Online

## Abstract

Little is known about the factors associated with engagement in mindfulness-based interventions (MBIs). Moreover, engagement in MBIs is usually defined in terms of class attendance (‘physical engagement’) only. However, in the psychotherapy literature, there is increasing emphasis on measuring participants’ involvement with interventions (‘psychological engagement’). This study tests a model that rumination and worry act as barriers to physical and psychological engagement in MBIs and that this in turn impedes learning mindfulness. One hundred and twenty-four participants were given access to a 2-week online mindfulness-based self-help (MBSH) intervention. Self-report measures of mindfulness, rumination, worry, positive beliefs about rumination, positive beliefs about worry and physical and psychological engagement were administered. A path analysis was used to test the linear relationships between the variables. Physical and psychological engagement were identified as two distinct constructs. Findings were that rumination and worry both predicted psychological disengagement in MBSH. Psychological engagement predicted change in the describe, act with awareness, non-judge and non-react facets of mindfulness while physical engagement only predicted changes in the non-react facet of mindfulness. Thus, rumination and worry may increase risk of psychological disengagement from MBSH which may in turn hinder cultivating mindfulness. Future suggestions for practice are discussed.

## Introduction

Mindfulness is a process of purposefully cultivating non-judgemental attention to experiences in the present moment (Kabat-Zinn 2003). Trait mindfulness is associated with increased subjective well-being and reduced psychological symptoms (Keng et al. 2011). Mindfulness-based interventions (MBIs) increase trait mindfulness, in turn resulting in psychological health benefits (Gu et al. 2015). Among the several interventions that have utilised this principle, mindfulness-based stress reduction (MBSR) and mindfulness-based cognitive therapy (MBCT) are two prominent psychological group-based interventions including primarily mindfulness practice and group discussion of principles (Baer 2003).

The effectiveness of MBIs for a range of mental health conditions is well established. There is evidence from multiple meta-analyses suggesting that MBIs have positive effects in clinical populations by, for example, reducing the relative risk of relapse in people who are currently well with a history of three or more episodes of depression (Piet and Hougaard 2011), reducing depressive symptom severity for people who are currently depressed (Strauss et al. 2014) and reducing anxiety symptoms (Khoury et al. 2013). In addition, MBIs can reduce stress in non-clinical populations (Chiesa and Serretti 2009).

Given the substantial evidence for effectiveness of group-based MBIs, research interest in mindfulness-based self-help (MBSH) interventions has proliferated as MBSH could provide a means of substantially widening access, particularly given some of the challenges with implementing MBCT in practice (Crane and Kuyken 2013). Mindfulness-based self-help leads to lower levels of depression and anxiety symptoms, at least in non-clinical populations (Cavanagh et al. 2013, 2014; Lever-Taylor et al. 2014). Evidence is also emerging that these benefits may extend to clinical populations. Dimidjian et al. (2014) trialled Mindful Mood Balance (MMB), a web-based MBCT program, with 100 people with a history of recurrent depression. There was a significant reduction in depressive symptom severity and rumination and a significant increase in self-reported mindfulness in participants of MMB compared to a non-randomised comparison group receiving usual care.

While much research has focused on the effectiveness of MBIs in improving psychological symptoms, measuring engagement in the intervention is also crucial. If engagement is poor, this will not only limit effectiveness but could also increase a sense of hopelessness for participants (Oei and Kazmierczak 1997), which in turn, may reduce psychological well-being (Fredrickson and Joiner 2002). Moreover, mindfulness involves decentering from the content of the thoughts and feelings. This ‘detached observation’ (Kabat-Zinn 1982, p. 34) of a constantly changing field is difficult to attain (Chambers et al. 2009) as the mind has a strong habitual tendency to wander to the content of thoughts. Additionally, direct engagement with negative thoughts during mindfulness practice might lead to an escalation of distress and a cycle of negative reinforcement (Bishop 2002). These potential negative consequences of mindfulness practice may result in disengagement from the practice (Lomas et al. 2014) and potentially to dropping out from the intervention.

Surprisingly, engagement in MBIs has not been clearly defined in the literature and there is lack of consensus on defining engagement in psychological therapies more broadly (Holdsworth et al. 2014; Tetley et al. 2011). A recent review of 79 studies of psychological therapies defined engagement in psychotherapy as ‘all the efforts that clients make during the course of treatment (both within and between sessions) toward the achievement of changes (treatment outcomes)’ (Holdsworth et al. 2014, p. 430). Engagement has been operationalized as a fourfold construct consisting of attendance, involvement, homework completion and therapeutic relationship (Holdsworth et al. 2014). It can be argued that attendance and homework completion measures the physical attributes of engagement, while involvement measures the psychological attributes of engagement. Involvement is described as including motivation, belief, commitment and intent to participate in the intervention (Holdsworth et al. 2014; Tetley et al. 2011). The construct of ‘involvement’ is particularly pertinent to MBIs as participating in MBIs is often described as involving ‘integrating mindfulness into life’ (Langdon et al. 2011, p. 276). The process of becoming more mindful appears to require ‘psychological participation’ (Kabat-Zinn 2003, p. 151) and involves not only performing discrete behaviour (e.g. ormal mindfulness practice) but also developing a radically different ‘being’ mode that can be entered at any time (Langdon et al. 2011). Thus, engaging in MBIs is perhaps somewhat different from engaging in other psychotherapies as mindfulness is often described as an ‘approach to life’ rather than a health behaviour (Langdon et al. 2011, p. 271).

We therefore propose a definition of engagement in MBIs. We suggest that engagement in MBIs involves *physical* engagement (session attendance and engagement in recommended between-session mindfulness practices) and *psychological* engagement. Psychological engagement that we propose consists of five factors: (1) *motivation* to put time aside to participate in the MBI course, (2) *intention* to maintain a personal formal mindfulness practice during and after the MBI course, (3) *commitment* to bringing mindfulness into daily life, (4) the *belief* that practicing mindfulness will be beneficial to one’s mental health or well-being and (5) the *therapeutic relationship* between the person and the MBI group and teacher. These five factors have established associations with treatment outcomes or treatment completion in the broader literature and so are good candidates to act as proxies for psychological engagement in MBIs: (1) motivation to participate in treatment is related to psychosocial functioning during treatment and to treatment progress (Simpson and Joe 2004), (2) intention is associated with treatment completion (Zemore and Ajzen 2014), (3) commitment or readiness is related to engagement in therapy (George et al. 1998), (4) belief in treatment effectiveness is associated with treatment retention (Kressel et al. 2000) and (5) the therapeutic relationship predicts attendance and participation in treatment (Lecomte et al. 2012).

We know surprisingly little about engagement in MBIs and its correlates. A recent meta-analysis of randomised controlled trials (RCTs) in clinical populations reported dropout from MBIs ranging from 8 to 38% (median = 15.5%) (Strauss et al. 2014). Another meta-analysis of RCTs reported mean dropout rates from MBSH interventions may typically be higher (37%) (Cavanagh et al. 2014), but similar to dropout rates in other self-help therapies (31%) (Melville et al. 2010). Another study reported that difficulties with emotion regulation, escape-avoidant coping and negative affect were all significantly associated with a failure to engage successfully in metacognitive acceptance training (Atkinson and Wade 2012). Only one published study to our knowledge has investigated predictors of physical engagement in MBIs (Crane and Williams 2010). An RCT of participants diagnosed with at least one episode of major depressive disorder (MDD) reported a 30% dropout from a face-to-face MBCT group (Crane and Williams 2010). In this study, participants with high levels of depressive rumination and brooding (i.e. facets of rumination) were more likely to drop out from the intervention. Although these conclusions were tentative due to the small sample size, the findings are theoretically meaningful.

Crane and Williams (2010) have argued that paradoxically, those who drop out from MBIs might be the very ones who could benefit the most had they engaged with the intervention. Moreover, qualitative studies have reported that participants may have difficulty in engaging in a mindfulness and meditation practices due to physical discomfort, feeling exhausted or disoriented, self-doubt and a feeling of being trapped in the long practices (Dobkin et al. 2012; Lomas et al. 2014). Thus, identifying factors associated with engagement in MBIs is crucial in order to enhance both physical and psychological engagement for those who may be most likely to benefit. Two variables that are likely to predict poor engagement in MBIs are perseverative thinking styles and positive beliefs about these thinking styles. Perseverative thinking styles, in particular rumination and worry, are antagonistic to the decentering processes involved in mindfulness (Wells 2005). Rumination has been defined as ‘repetitive and passive thinking about one’s symptoms of depression and the possible causes and consequences of these symptoms’ (Nolen-Hoeksema 2003, p. 107), while worry has been defined as ‘a chain of thoughts and images, negatively affect-laden and relatively uncontrollable … [that] represents an attempt to engage in mental problem-solving on an issue whose outcome is uncertain but contains the possibility of one or more negative outcomes’ (Borkovec et al. 1983, p. 10). Rumination and worry are closely related processes. They both involve abstract-level thinking about problems, are typically experienced as uncontrollable and have negative consequences—rumination and worry are, respectively, implicated in the maintenance of depression and generalized anxiety disorder (Kertz et al. 2015), However, there are also differences between the two. Rumination is typically seen as past or present focused, whereas worry is typically seen as future focused (Nolen-Hoeksema et al. 2008). It is also suggested that worry involves a greater degree of verbal thought than rumination and that, conversely, rumination involves a greater degree of imagery than worry (Papageorgiou and Wells 2003). Therefore, while closely related processes, rumination and worry are sufficiently distinct to warrant separate examination.

People who tend to ruminate and/or worry may find that they struggle to decentre during mindfulness practice and instead get lost in rumination or worry, heightening their distress and leading them to believing that mindfulness is unhelpful and then dropping out (as was found in the study by Crane and Williams 2010). Furthermore, the metacognitive model of emotional vulnerability suggests that perseverative negative thinking, such as depressive rumination and anxious worry, is associated with metacognitive beliefs about the functions and consequences of such thinking (Wells and Matthews 1996). In addition, ruminating and worrying may result in reinforced positive beliefs about rumination and worry. If people believe that rumination and worry help them to solve the problem that they are ruminating/worrying about and/or will help them to prevent the worried-about event from coming true, they may not believe that decentring from and letting go of difficult thoughts will be helpful leading to disengagement from the MBI.

MBSHs are likely to be particularly effective ways of studying engagement in MBIs as MBSHs remove many of the non-specific factors in face-to-face MBIs that may themselves enhance engagement such as support from the group members and mindfulness teacher. The current study tests a model of engagement in online MBSH. Based on existing research and theory, we hypothesise that (1) baseline levels of perseverative thinking (rumination and worry), and baseline positive beliefs about rumination and worry, will predict physical and psychological disengagement with MBSH; and (2) greater physical and psychological engagement in MBSH will in turn be associated with improvement in each facet of trait mindfulness. Additionally, we explore the association between physical and psychological engagement in MBIs.

## Method

### Participants

Jackson (2003) suggested that the sample size to parameter ratio of 20:1 is ideal and 10:1 is acceptable for path analysis. As dropout rates from MBSH interventions can be quite high (mean reported dropout 37%; see Cavanagh et al. 2014), a total of 124 participants were recruited in order to achieve a completer data set within this range. Participants were recruited to the study from a university in the South of England by responding to emails or posters advertising the study. Ethical approval was obtained from the host university ethics committee. All participants recruited for the study provided written consent to take part in the study. Participants were advised of the difficult feelings that may arise due to mindfulness practice and their right to self-exclude themselves from the study if such feelings arose. Participants were also advised to contact the research team and the university counselling services in such circumstances. None of the participants who took part in the study contacted the research team regarding difficulties arising due to mindfulness practice. Age ranged from 18 to 61 years (*M* = 23.4 years, SD = 6.6 years), 76% were female, 83% were of white ethnicity, 84% were current students while the rest were current staff and 72% had no prior experience of mindfulness.

### Procedure

Participants were given the link to the baseline questionnaires (hosted by http://www.surveymonkey.com). On completion, access to the Learning Mindfulness Online (LMO) intervention (Cavanagh et al. 2013) site through the university’s virtual learning environment was provided. After the 14-day intervention period, participants were sent the post-intervention questionnaire link.

#### Intervention

The LMO intervention (Cavanagh et al. 2013) comprised of six sections. The *Welcome* page was followed by the *Daily Mindfulness Practice* page, which included a choice of male and female voices for a 10-min guided mindfulness meditation practice. The other pages included information on *Everyday Mindfulness Activities* (such as mindful tooth brushing and eating); *Daily Practice and Everyday Mindfulness Activities FAQ* (including information on range of emotions and feelings, both good and bad that may result from mindfulness practice); *My Daily Journal* (providing opportunity to record participants’ thoughts and feelings as they progress through the intervention) and *Help and Assistance.* The section on *Study Information* provided crucial information regarding participation in the study along with contact details of the researchers and university counselling services (see Cavanagh et al. 2013 for details). Participants were given access to the program for 14 days and were invited to practice mindfulness at least once a day during these 14 days.

### Measures

#### Five Facet Mindfulness Questionnaire – Short Form (Bohlmeijer et al. 2011)

The Five Facet Mindfulness Questionnaire – Short Form (FFMQ-SF) is a 24-item self-report scale, with each item rated on a 1 to 5 scale, where 1 is never or very rarely true and 5 is very often or always true. It assesses five facets of mindfulness: observing, describing, acting with awareness, non-judging and non-reactivity, and subscales have good internal consistency in this study (*α* = 0.89). However, a recent hierarchical confirmatory factor analysis revealed that in a non-meditative sample, a four-factor FFMQ (FFMQ minus the ‘observe’ subscale) is preferred over a five-factor score (Gu et al. 2016). Hence, four facets (describing, acting with awareness, non-judging and non-reactivity) of the scale will be included in the analysis.

#### Ruminative Response Subscale (Nolen-Hoeksema and Morrow 1991)

The Ruminative Response Subscale (RRS) is a subscale of the response styles questionnaire (RSQ; Nolen-Hoeksema and Morrow 1991) with 22 items, each item rated on a 4-point scale, where 1 is almost never and 4 is almost always. Participants were asked to assess how each item applied to them when they feel down, sad or depressed. The internal consistency in this study (Cronbach’s *α*) is 0.95.

#### Penn State Worry Questionnaire (Meyer et al. 1990)

The Penn State Worry Questionnaire (PSWQ) consist of 16 items, each rated on a 5-point scale, where 1 is not at all typical of me and 5 is very typical of me. Participants were asked to rate how each item were typical of them. The PSWQ had moderate internal consistency in this study (*α* = 0.70).

#### Positive Beliefs About Rumination Scale (Papageorgiou and Wells 2001)

The Positive Beliefs about Rumination Scale (PBRS) consists of nine items, each rated on a 4-point scale from 1—do not agree to 4—agree very much, and assesses positive beliefs about rumination. Participants were asked to respond how much they generally agree with each on the statements in the questionnaire. The PBRS has high internal consistency (*α* = 0.88 in this study).

#### Positive Beliefs About Worry (Wells and Cartwright-Hatton 2004)

The positive beliefs about worry (PBAW) was measured using a subscale of the metacognitions questionnaire (MCQ) called *positive* beliefs and consisted of six items measured on a 4-point scale, where 1 is do not agree and 4 is agree very much. Participants were invited to respond how much they generally agreed with the beliefs listed in the questionnaire. The subscale had high internal consistency (*α* = 0.88).

#### Measures of Engagement

The two engagement scales are free to use by future research studies by citing this paper at no cost. Formatted copies of the scales are available from the corresponding author. Measures of engagement were reported at a post-intervention only.

##### Physical Engagement

Physical engagement was defined as the frequency of mindfulness practice and this was measured using two self-report questions. The items were ‘on how many days [over the past two weeks] did you practice mindfulness meditation at least once?’ and ‘how many times on an average did you practice mindfulness meditation each day?’. The total physical engagement score was calculated by multiplying these two figures together (i.e. physical engagement = number of days on which mindfulness was practiced × number of times per day mindfulness was practice).

##### Psychological Engagement

An existing validated measure of psychological engagement in MBIs could not be found and therefore a measure was developed for this study. Items were developed to measure each of the elements in our proposed definition of psychological engagement in MBIs (see above), but without an item for the ‘therapeutic relationship’ element given, this is a pure self-help intervention. This resulted in a four item measure: (1) Motivation (*[over the past two weeks]*, *how motivated were you to set time aside to use the mindfulness online course?*)*,* (2) Intent (*how likely do you think you are to engage in mindfulness?*), (3) Commitment (*how often did you bring mindfulness principles into your life each day*?) and (4) Belief (*how effective do you think mindfulness is in helping to deal with stressful situations?*). Items were rated on a 5-point scale ranging from 1 (not at all) to 5 (completely). This scale had high internal consistency (*α* = 0.82).

### Data Analyses

Data were analysed using SPSS (Windows version 22.0) and AMOS Graphics (version 22.0; Arbuckle 2006) software. As a first step, correlations between all the variables were examined. Model fit was evaluated using several fit indices and convergence between findings was assessed (Byrne 2010), namely, the Satorra-Bentler chi-square, the root mean square error approximation (RMSEA), the goodness of fit index (GFI) and the comparative fit index (CFI). The Satorra-Bentler chi-square is a chi-square fit index that corrects the statistic under distributional violations by determining whether the value of this statistic is less than twice the model’s degrees of freedom (Kline 2005). Chi-square is influenced by the sample size. To address this issue, the chi-square value was divided by the degrees of freedom (df). A value below of *χ* ^2^/df below five indicates acceptable model fit and a value close to two indicates a good fit. Second, RMSEA values of 0.08 and 0.05 are interpreted to reflect acceptable and good model fit, respectively. The closer the value of GFI index is to 1, the better the fit. The CFI measures the proportional improvement in fit by comparing a hypothesised model with a more restricted baseline model. The CFI indexes also range from 0 (absolute lack of fit) to 1 (perfect fit). A CFI value larger than 0.90 indicates an adequate model fit.

## Results

Of one hundred and twenty-four participants who agreed to take part in the study, 81 (65%) participants completed the post-intervention questionnaires. Among the participants who completed the post-intervention measures, five participants did not complete the engagement questions and so were excluded from the analysis. This resulted in 76 participants being included in the analysis (61% of original sample). The mean age of these participants was 24.65 years (SD = 7.67, range = 18–61); 75% of completers were female. There were no significant differences in age, gender, role in university, rumination, worry, positive beliefs about rumination and worry and mindfulness between study completers and non-completers.

Descriptive statistics and correlation coefficients between path analysis variables are shown in Table 1. This shows that baseline rumination and worry were both associated with poorer physical engagement and psychological engagement at the zero-order level. Positive beliefs about rumination and positive beliefs about worry on the other hand were not significantly associated with measures of engagement. Finally, there was a significant correlation between physical and psychological engagement but with only a small medium effect size suggesting that these variables may be partially independent.

**Table 1:** Descriptive statistics (range, means and standard deviation) and Pearson’s correlation coefficients of the measures (*N* = 76)

		Mean (SD)	Range	1	2	3	4	5	6	7	8	9	10
1	Baseline describe	16.87 (3.26)	10–24	–									
2	Baseline act aware	14.81 (3.89)	5–24	0.37**	–								
3	Baseline non-judge	15.25 (3.85)	7–24	0.40**	0.44**	–							
4	Baseline non-react	14.24 (3.29)	5–22	0.34**	0.49**	0.53**	–						
5	Baseline RRS	51.03 (16.38)	22–79	− 0.02	− 0.05	− 0.24*	− 0.1	–					
6	Baseline PSWQ	46.82 (10.21)	25–64	0.05	− 0.03	− 0.19	− 0.05	0.38**	–				
7	Baseline PBRS	24.39 (5.67)	13–36	− 0.15	− 0.14	− 0.33*	− 0.25*	0.07	− 0.21	–			
8	Baseline PBAW	10.50 (3.97)	6–24	− 0.18	− 0.12	− 0.26*	− 0.26**	0.12	0.15	0.38*	–		
9	Physical engagement	5.61 (1.86)	1–12	− 0.04	0.12	0.08	0.05	− 0.64**	− 0.33**	0.01	0.08		
10	Psychological engagement	3.21 (.77)	2–4.5	− 0.12	− 0.08	− 0.01	− 0.06	− 0.40**	− 0.43**	0.17	0.15	0.29**	–

Baseline describe, act aware, non-judge and non-react measured by Five Facet Mindfulness Questionnaire – Short Form (Bohlmeijer et al. 2011)

*PBAW*, positive beliefs about worry (Wells and Cartwright-Hatton 2004); *PBRS*, Positive Beliefs about Rumination Scale (Papageorgiou and Wells 2001); *PSWQ*, Penn State Worry Questionnaire (Meyer et al. 1990); *RRS*, Ruminative Response Subscale (Nolen-Hoeksema and Morrow 1991)

*p < 0.05

**p < 0.01

### Engagement in the MBSH Intervention

See Table 1 for the means and standard deviations on the physical and psychological engagement measures. Further details are that 77% of participants reported practicing mindfulness once per day during the intervention period while 20% participants practised mindfulness more than once a day. In terms of the psychological engagement questionnaire, levels of engagement (indicating 3 or more on the subscale) were as follows: 84.2% of participants said that they were motivated to set time aside to use the mindfulness online course, 68.4% participants said that they were likely to engage in mindfulness practice, 57.8% participant brought mindfulness principles into their daily life each day and 79.0% of participants reported that mindfulness was effective in helping them deal with stressful situations.

### Path Analysis Findings

**Fig. 1:**
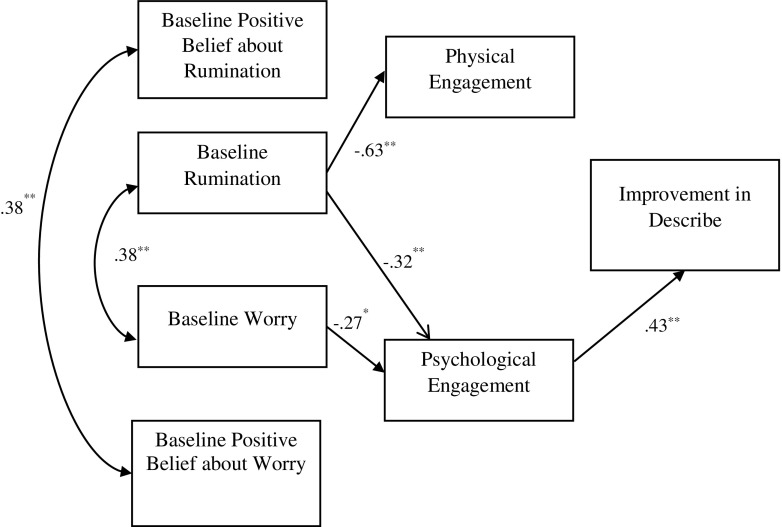
Empirical model for improvements in the ‘describe’ FFMQ facet, showing significant standardised path coefficients. Double-pointed arrows depict covariance. *N* = 76; **p < 0.001

**Fig. 2:**
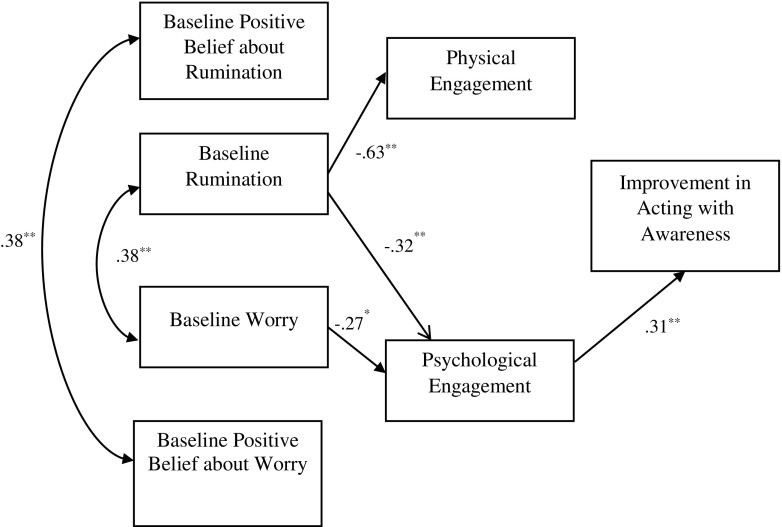
Empirical model for improvements in the ‘acting with awareness’ FFMQ facet, showing significant standardised path coefficients. Double-pointed arrows depict covariance. *N* = 76; **p < 0.001

**Fig. 3:**
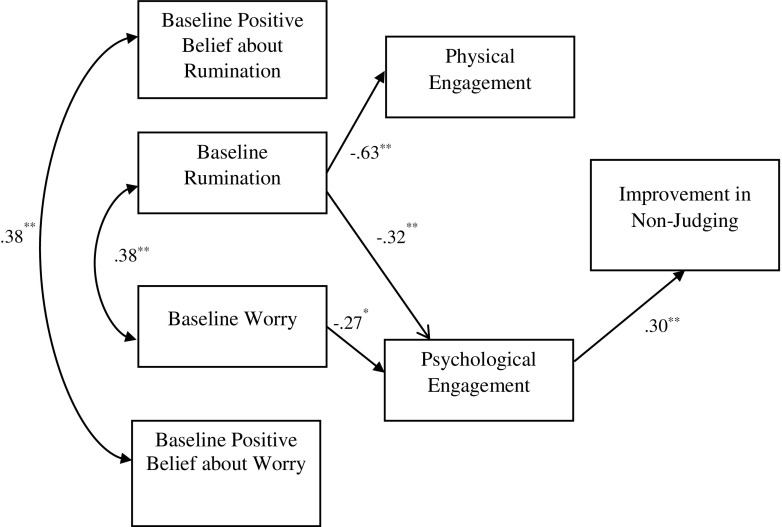
Empirical model for improvements in the ‘non-judge’ FFMQ facet, showing significant standardised path coefficients. Double-pointed arrows depict covariance. *N* = 76; **p < 0.001

**Fig. 4:**
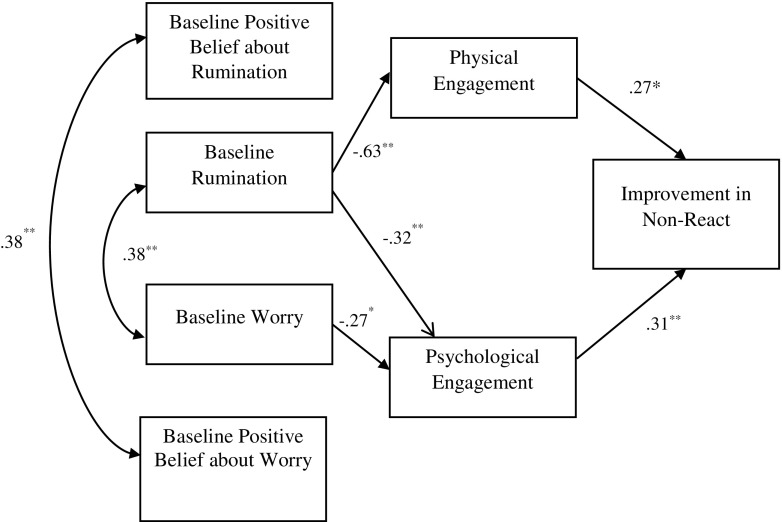
Empirical model for improvements in the ‘non-react’ FFMQ facet, showing significant standardised path coefficients are presented. Double-pointed arrows depict covariance. *N* = 76; **p < 0.001

Each of the facets of FFMQ (all minus ‘observe’) was tested in separate models (Figs. 1, 2, 3 and 4). Seven observable variables were tested in each of the models. The models showed non-significant paths between worry and physical engagement (*β* = − 0.07, *p* = 0.49), positive beliefs about worry and physical (*β* = 0.15, *p* = 0.11) and psychological (*β* = 0.11, *p* = 0.30) engagement, positive beliefs about rumination and physical (*β* = − 0.02, *p* = 0.82) and psychological (*β* = − 0.09, *p* = 0.37) engagement. The paths between physical engagement and change in ‘describing’ (*β* = − 0.09, *p* = 0.42) (Fig. 1), ‘acting with awareness’ (*β* = 0.12, *p* = 0.29) (Fig. 2) and ‘non-judging’ (*β* = − 0.04, *p* = 0.71) (Fig. 3) subscales were also not significant.

The model fit statistics from testing the four models are shown in Table 2. Model 1 (including the ‘describing’ facet) showed a good fit with the data, while models 3 (‘non-judging’) and 4 (‘non-react’) showed an acceptable fit. The RMSEA index of model 2 (‘act with awareness’) indicated a poor fit. The empirically supported models as well as standardised coefficients and *R* ^2^ values are shown in Figs. 1, 2, 3 and 4, with *R* ^2^ values shown above each endogenous variable. Rumination yielded statistically significant path coefficients to physical engagement (explaining 63% of the variance of this variable), with higher levels of rumination related to lower levels of physical engagement and to lower levels of psychological engagement (explaining 32% of the variance). Worry on the other hand only yielded a statistically significant path coefficient to psychological engagement (explaining 28% of the variance). In addition, significant paths were noted between psychological engagement and improvement in describe (explaining 43% of the variance), acting with awareness (explaining 31% of variance), non-judge (explaining 30% of the variance) and non-react (explaining 31% of variance). Physical engagement had significant path with non-react (explaining 27% of variance).

**Table 2:** Fit indices for the empirically derived path models

Model	*χ* ^2^ (df)	*χ* ^2^ ÷(df)	RMSEA (90% CI)	GFI	CFI
Model 1 (describe)	5.38 (5)	**1.08**	**0.03** (0.00–0.17)	**0.98**	**0.99**
Model 2 (acting with awareness)	9.18 (5)	**1.84**	0.11 (0.00–0.21)	**0.97**	**0.95**
Model 3 (non-judging)	7.29 (5)	**1.46**	*0.08* (0.00–0.19)	**0.97**	**0.98**
Model 4 (non-reactivity)	7.27 (5)	**1.45**	*0.08* (0.00–0.19)	**0.97**	**0.98**

Bold indices indicate good model fit, and indices with italics indicate an acceptable fit. Root mean square error of approximation (RMSEA), goodness of fit index (GFI), adjusted GFI (AGFI) and comparative fit index (CFI) > 0.9 indicate adequate fit

## Discussion

The primary aim of the study was to test theoretically defined models of engagement in MBIs and this was partly supported empirically in the final models (Figs. 1, 2, 3 and 4). It is of note that the association between physical and psychological engagement was small medium in size supporting the assertion that these are partially independent constructs. The final models show that baseline worry and rumination were both associated with poor psychological engagement in the MBSH intervention. Baseline rumination, but not worry, was associated with poor physical engagement in MBSH. Contrary to hypotheses, beliefs about worry and rumination did not play a part in the final model as these variables were not associated with either physical or psychological engagement. Interestingly, psychological engagement was associated with pre-post MBSH improvements in all the four facets of mindfulness (describing, acting with awareness, non-judging and non-react), while physical engagement was only associated with improvement in non-judgement.

As predicted, our study showed that trait rumination and worry prior to starting the MBSH intervention were related to psychological engagement in the intervention, with rumination also associated with physical engagement. This is consistent with the findings of a previous study (Crane and Williams 2010) that found that participants who dropped out from MBIs had higher levels of depressive rumination and brooding at baseline than those not dropping out. Rumination and worry are habitual and relatively stable perseverative thinking styles (Watkins 2008), are typically experienced as uncontrollable and are implicated in the maintenance of depression and generalized anxiety disorder, respectively (Kertz et al. 2015). It is likely therefore that when first beginning to engage in mindfulness practice, people who tend to ruminate and worry will find that they get absorbed in rumination and worry, potentially leading to lower mood and/or heightened anxiety. Of course, the hope is that with continued mindfulness practice, people will acquire the skills to notice the mind wandering to rumination or worry and to choose to bring the mind to a different point of attention, short-circuiting these perseverative thinking processes and curtailing their negative effects. Indeed, MBIs have beneficial effects on mental health outcomes through, in part, reducing worry and rumination (Gu et al. 2015). However, negative experiences of mindfulness practice in the early stages of MBIs may lead people to disengage, preventing the potential benefits that may come with longer-term practice and inhibiting the development of positive beliefs about mindfulness. The struggle with habitual perseverative thinking coupled with a lack of belief in mindfulness may, understandably, result in disengagement from MBIs.

The finding that rumination and worry are associated with psychological disengagement from MBSH presents a challenge to the dissemination of mindfulness teaching via self-help in particular but may be also relevant to face-to-face MBIs (see Crane and Williams 2010). Rumination and worry mediate depression and anxiety (Muris et al. 2005), and there is substantial evidence suggesting that MBIs are effective in the treatment of depression and anxiety (Hofmann et al. 2010) with effects mediated by reductions in rumination and worry (Gu et al. 2015). Hence, the people who might benefit the most from MBIs are the very ones who are most likely to disengage from the intervention. Addressing this issue of disengagement is crucial in ensuring that the reach of potential benefits of MBIs can be extended to the people who could benefit the most.

Interestingly, rumination and not worry predicted physical engagement. While this is an early-stage study and findings require replication, if this finding is repeated, it could indicate that rumination during mindfulness practice is experienced as particularly aversive, leading people who tend to ruminate to disengage from mindfulness practice more readily than people who tend to worry. This would be a particular concern given the key role that rumination plays in the onset and maintenance of depression (Nolen-Hoeksema et al. 2008), the mental health condition with arguably the strongest evidence for the effectiveness of MBIs (Kuyken et al. 2016; Strauss et al. 2014).

It is of interest that effects on cultivating mindfulness were evident for psychological engagement over and above that for physical engagement. Indeed, only psychological engagement was associated with pre-post MBSH improvements in all the four facets of mindfulness (describing, act with awareness, non-judge and non-react). This fits with the suggestion made earlier that psychological engagement in MBIs may be particularly important in determining benefits, and this may be over and above the importance of physical engagement. The suggestion that mindfulness requires ‘psychological participation’ (Kabat-Zinn 2003, p. 151) and is an ‘approach to life’ rather than a health behaviour (Langdon et al. 2011, p. 271) is relevant here. Our findings are consistent with the suggestion that a tendency to ruminate or worry leads to psychological disengagement in MBSH (poor motivation, intent, commitment and belief) which in turn leads to reduced improvements in all facets of trait mindfulness, although these causal hypotheses require testing in future research.

Interestingly, only physical engagement in the intervention was associated with change in the non-react facet of mindfulness. This finding is similar to previous findings that have reported that formal home practice is significantly associated to outcomes such as lower risk of relapse to depression in MBCT (Crane et al. 2014; Hawley et al. 2014; Perich et al. 2013). However, it is unclear why this association was not noted for other facets of mindfulness. One possible explanation could be that in the current non-clinical population, regular physical engagement to mindfulness meditation is not required to develop metacognitive insight of mindfulness. However, this suggestion also needs to be interpreted with caution as it is only reflective of a non-clinical population and a brief, 2-week MBSH intervention. Future studies can investigate whether physical or psychological engagement have differential associations on the beneficial effects of standard MBIs (i.e. MBCT/MBSR) in clinical populations. Nevertheless, the findings clearly highlight the value of measuring psychological engagement in MBIs rather than simply quantifying engagement as the number of classes attended or amount of mindfulness practice engaged in.

Another interesting finding is the low shared variance between physical and psychological engagement with these variables sharing only around 5% of their variance. This suggests that psychological engagement in mindfulness is not closely associated with amount of mindfulness practice. Psychological participation in the MBSH intervention was associated with increased mindfulness over the course of the intervention while physical engagement was not. This is contrary to evidence that suggests that amount of mindfulness practice may be associated with greater increases in mindfulness (Carmody and Baer 2008). Teasdale (1999) identified two distinct types of metacognition in relation to MBIs, namely, metacognitive knowledge and metacognitive insight. Metacognitive knowledge may be defined as *knowing* that thoughts are not always accurate while metacognitive insight is *experiencing* thoughts as events (Teasdale 1999). Since there are some similarities between our constructs of physical and psychological engagement and metacognitive insight and knowledge, our findings could suggest that *knowing* mindfulness skills and *experiencing* these may only be loosely related to each other and that it may be possible to develop one without the other. Moreover, our findings are consistent with the suggestion that metacognitive knowledge may be particularly important in determining improvements in mindfulness in MBSH. Future studies can investigate whether physical or psychological engagement have differential associations in enhancing mindfulness in standard MBIs (MBCT/MBSR) and in clinical populations. Since it is imperative to physically engage in mindfulness practice to some extent in order to be psychologically involved with mindfulness, future research could also explore the extent of mindfulness practise required to develop metacognitive knowledge of mindfulness and be psychologically engaged without physical engagement.

Contrary to our hypothesis, positive beliefs about rumination and worry at baseline were not associated with either physical or psychological engagement in the MBSH intervention and effect sizes were negligible (i.e. these findings are unlikely due to low statistical power). Moreover, we found that these positive beliefs did not correlate with rumination or worry at baseline. While this is contrary to some previous findings (Papageorgiou and Wells 2004; Watkins and Baracaia 2001), recent evidence suggests that positive beliefs about rumination and worry may not be associated with depression and anxiety (Gawęda and Kokoszka 2014), questioning their role in these conditions. The lack of association in the current study between these positive beliefs and rumination and worry highlights the need for further research into these constructs and the role that they may or may not play in causing and maintaining depression and anxiety.

### Limitations

There were a number of limitations with this study. First, the sample predominantly consisted of young adults from a single university; this may restrict the generalizability of the findings of this study. Second, the measure of psychological engagement was developed for this study. This scale has face validity to the definition of engagement that we have tested, has initial evidence of convergent validity as psychological engagement was strongly associated with improvement in trait mindfulness and has high internal consistency (*α* = 0.82). However, the validity of the scale has not been established. Moreover, the construct of psychological engagement in MBIs may not be comprehensive, and future research could also explore any additional factors that may constitute psychological engagement specifically in MBIs. Also, in our measure of psychological engagement, we did not include an item to tap the ‘therapeutic relationship’ element as ours was a pure self-help intervention. However, there is emerging evidence that people can develop a meaningful relationship with self-help interventions (Cavanagh and Millings 2013) through the relationship built between the reader and the author (e.g. how we imagine the author to be, feeling understood by them). Third, the accuracy of physical engagement reported by participants was not assured. Physical engagement scores (i.e. time spent in mindfulness practice) may be influenced by social desirability effects, as with any self-report measures. In addition, psychological engagement could be overestimated when reported by the participants due to factors such as social desirability and retrospective self-report bias. Future studies should test these possible confounds and technology could be used to objectively monitor level of engagement in the online intervention. Fourth, our results directly apply to online MBSH only. However, MBSH is a particularly good test of engagement in MBIs as it removes many of the non-specifics of MBIs that may enhance engagement such as other group members and a supportive mindfulness teacher. Fifth, the rate of attrition was high in this study. This rate is similar to other studies that have used an online self-help-based mindfulness intervention (see Cavanagh et al. 2014). No significant differences were noted among study completers and non-completers. In addition, since the final sample consisted of only 76 participants, this could reduce the power of the study resulting in non-significant paths. Given the high rate of attrition, future studies could also explore the reasons of dropping out of the study. Additionally, this study examined the effect on each facet of mindfulness using four different models due to the small sample size. Since each of these facets may be correlated, future studies should examine a model including all the different mindfulness facets in one model. Moreover, the model for the ‘act awareness’ facet did not show acceptable fit in all the indices; hence, the model estimates for this facet may be less reliable. Finally, mindfulness research is often hampered due to the problems of measuring mindfulness itself. Measurement of mindfulness is dependent on a self-report measure in this study, but at present, there are no well-established reliable and valid alternatives (Bergomi et al. 2013).

### Future Directions

This study has identified factors that are related to poor engagement in MBSH—namely rumination and worry. Similar associations have also been identified for face-to-face MBCT (Crane and Williams 2010), and it may be useful for both self-help and face-to-face MBIs to incorporate more discussion and psychoeducation on how rumination and worry might present challenges to practice and how to respond to this. This could include support with ways of responding to distressing thoughts and feelings during mindfulness practice and the rationale for the intervention in terms of reducing worry and rumination, and that this may take some practice. As an extension of the current findings, future research could address the question of whether these models can also predict more distal outcomes such as symptom measures.

Another interesting finding in this study is the differential associations of physical and psychological engagement to outcomes in MBIs. According to the MBSR and MBCT protocols, participants attending at least four classes are classed as having ‘completed’ the intervention. However, attending classes may not always correspond to psychological participation in the intervention (as demonstrated by the small medium-sized correlation in this study between psychological and physical engagement). Hence, future research could investigate the effects of both forms of engagement in MBIs.

Finally, an attempt to cultivate positive beliefs about mindfulness could be incorporated into the online MBSH intervention used in this study in order to increase psychological engagement in the intervention. Changes in the program content by including more psychoeducation and interactive elements may result in increased positive beliefs about mindfulness (and thereby increasing psychological engagement) and potentially to increases in mindfulness and associated benefits to psychological health. Future research could explore the differential effect of MBIs with or without psychoeducation.

In summary, this study tested four path analysis models and found that baseline rumination and worry were associated with poorer psychological engagement in MBSH and psychological engagement was associated with all the four facets of mindfulness while physical engagement was only associated with the ‘non-react’ facet of mindfulness. This is despite the fact that people with high trait rumination or worry might be the very people who might benefit most. Furthermore, two facets of engagement (physical and psychological) in mindfulness-based interventions (MBIs) were identified and results suggested that these two facets of engagement are partially independent. Findings pave the way for future research exploring ways of optimising engagement in MBSH specifically but also in MBIs more generally and in particular optimising engagement for those people who might benefit most.
